# Short open reading frames (sORFs) and microproteins: an update on their identification and validation measures

**DOI:** 10.1186/s12929-022-00802-5

**Published:** 2022-03-17

**Authors:** Alyssa Zi-Xin Leong, Pey Yee Lee, M. Aiman Mohtar, Saiful Effendi Syafruddin, Yuh-Fen Pung, Teck Yew Low

**Affiliations:** 1 UKM Medical Molecular Biology Institute (UMBI), Universiti Kebangsaan Malaysia, 56000 Kuala Lumpur, Malaysia; 2 Division of Biomedical Science, School of Pharmacy, University of Nottingham Malaysia, Semenyih, 43500 Selangor, Malaysia

**Keywords:** Short open reading frame (sORF), Small open reading frame (smORF), Microproteins, Ribosome profiling (RIBO-Seq), Mass spectrometry, Proteogenomics

## Abstract

A short open reading frame (sORFs) constitutes ≤ 300 bases, encoding a microprotein or sORF-encoded protein (SEP) which comprises ≤ 100 amino acids. Traditionally dismissed by genome annotation pipelines as meaningless noise, sORFs were found to possess coding potential with ribosome profiling (RIBO-Seq), which unveiled sORF-based transcripts at various genome locations. Nonetheless, the existence of corresponding microproteins that are stable and functional was little substantiated by experimental evidence initially. With recent advancements in multi-omics, the identification, validation, and functional characterisation of sORFs and microproteins have become feasible. In this review, we discuss the history and development of an emerging research field of sORFs and microproteins. In particular, we focus on an array of bioinformatics and OMICS approaches used for predicting, sequencing, validating, and characterizing these recently discovered entities. These strategies include RIBO-Seq which detects sORF transcripts via ribosome footprints, and mass spectrometry (MS)-based proteomics for sequencing the resultant microproteins. Subsequently, our discussion extends to the functional characterisation of microproteins by incorporating CRISPR/Cas9 screen and protein–protein interaction (PPI) studies. Our review discusses not only detection methodologies, but we also highlight on the challenges and potential solutions in identifying and validating sORFs and their microproteins. The novelty of this review lies within its validation for the functional role of microproteins, which could contribute towards the future landscape of microproteomics.

## Background

Spurred by the first draft of the human genome in 2001, the number of annotated human genes increased drastically in the next decade, with ~ 20,000 papers published annually on the protein-coding genes [1, 2]. By decoding the bulk of human DNA, the Human Genome Project (HGP) had empowered genomic research and extended its impacts to many other species including mouse, rat, fruit-fly and even to plants such as *Arabidopsis thaliana*.

Genome annotation refers to the procedure whereby the locations, coding regions and functions of genes are determined within a sequenced genome. This process is typically performed by automated bioinformatic pipelines based on (i) the innate characteristics and features of genomic sequences, as well as (ii) the sequence homology conserved through evolution [3, 4]. These two principles tie together the possibility of genes acquiring evolutionary selective advantages, thus producing proteins with functionalities essential for survival. Via this process, putative protein-coding genes can be predicted ab initio. This kickstarted the search for protein-coding genes, plateauing in the mid-2000s at ~ 20,000 protein-coding genes for the human genome [2]. However, this number may be underestimated due to an arbitrary limitation of a 300-base (100-codon) cut-off for transcripts. Such restrictions are imposed on ORF-prediction algorithms by the Functional ANnoTation Of the Mammalian Genome (FANTOM) consortium to minimize false positive predictions, especially the mis-classification of non-coding RNAs as mRNAs [5–8].

In the past two decades, the scientific community had nonetheless found mounting experimental evidence for open reading frames (ORFs) comprising < 100 codons. These so-called short ORFs (sORFs) or small ORFs (smORFs) can encode functional and stable sORF-encoded protein (SEPs) or microproteins. The first revelation of a functional microprotein came from the discovery of a novel helix-loop-helix protein in 1990 by Benezra et al., known as the “Id” protein. Id functions by inhibiting the trans-activation of MCK gene at the MyoD consensus binding site in myoblasts during muscle differentiation [9]. Whereas for plants, the first discovered microproteins are the LITTLE ZIPPER (ZPR) proteins. Being functional analogues to “Id” proteins, ZPR proteins control stem cell maintenance during plant development [10].

Several recently identified microproteins have been associated with diseases. For example, a 54-amino acid mitochondrial microprotein known as PIGBOS was believed to play a role in stress signalling. PIGBOS is localised in the mitochondrial membrane and mediates signalling events leading to the unfolded protein response (UPR) [11]. This microprotein interacts with CLCC1, an endoplasmic reticulum (ER) protein, forming a connection between the mitochondria and the ER [12]. PIGBOS’s role in stress signalling may indicate a new direction in research concerning high ER stress, including cancer. Another microprotein that is linked to cancer is CYREN, which inhibits non-homologous end joining (NHEJ) repair during S and G2 phases [13]. CYREN ensures accurate homologous recombination, and its dysfunction may destabilise DNA integrity.

In accordance with advances in OMICS research and bioinformatics, functional and stable microproteins have increasingly been identified and characterized. We therefore dedicate this review to discuss the current developments in microproteome research, followed by state-of-the-art strategies that are used to identify and validate them at different biomolecular levels.

## Short open reading frames (sORFs)

Conventionally, an ORF is defined as a stretch of consecutive and non-overlapping nucleotide triplets (codons) that can be translated into proteins, whereby it should also initiate with an in-frame start codon (AUG), and terminate with one of the three stop codons (UAA, UAG, UGA). In a theoretical manner, Olexiouk et al. (2018) estimated that the probability of randomly generating a start codon within the nucleotide space is 1 out of 64, and that the chances of finding a stop codon within the next 99 codons is ~ 99%; discounting splice variants, reading frames and GC-rich regions, strandedness and nucleotide biases [14]. Consequently, this means that ~ 1.5% of the genome may encode ORFs < 100 codons [14, 15]. Naturally, this results in an unreasonably large number of putative sORFs, whose chances of being transcribed and translated into functional polypeptides seem far-fetched. Hence, a 300-nucleotides cut-off was introduced, as most of these sORFs were deemed meaningless and random [16, 17].

Another contributing factor for this cut-off is that existing algorithms are so far not ideal for annotating the sORFs. This is due to the propensity of short sequences to score low in evolutionary conservation, an indicator for functionality [18]. Combined with the technical difficulties in delineating sORFs from chance in-frame start and stop codons and to isolate sORF-translated microproteins, these sORFs were either considered noise, occurring by chance, non-coding or unlikely to be translated [19–21]. As such, these sORFs and the resulting microproteins were traditionally ignored by the scientific community.

**Fig. 1:**
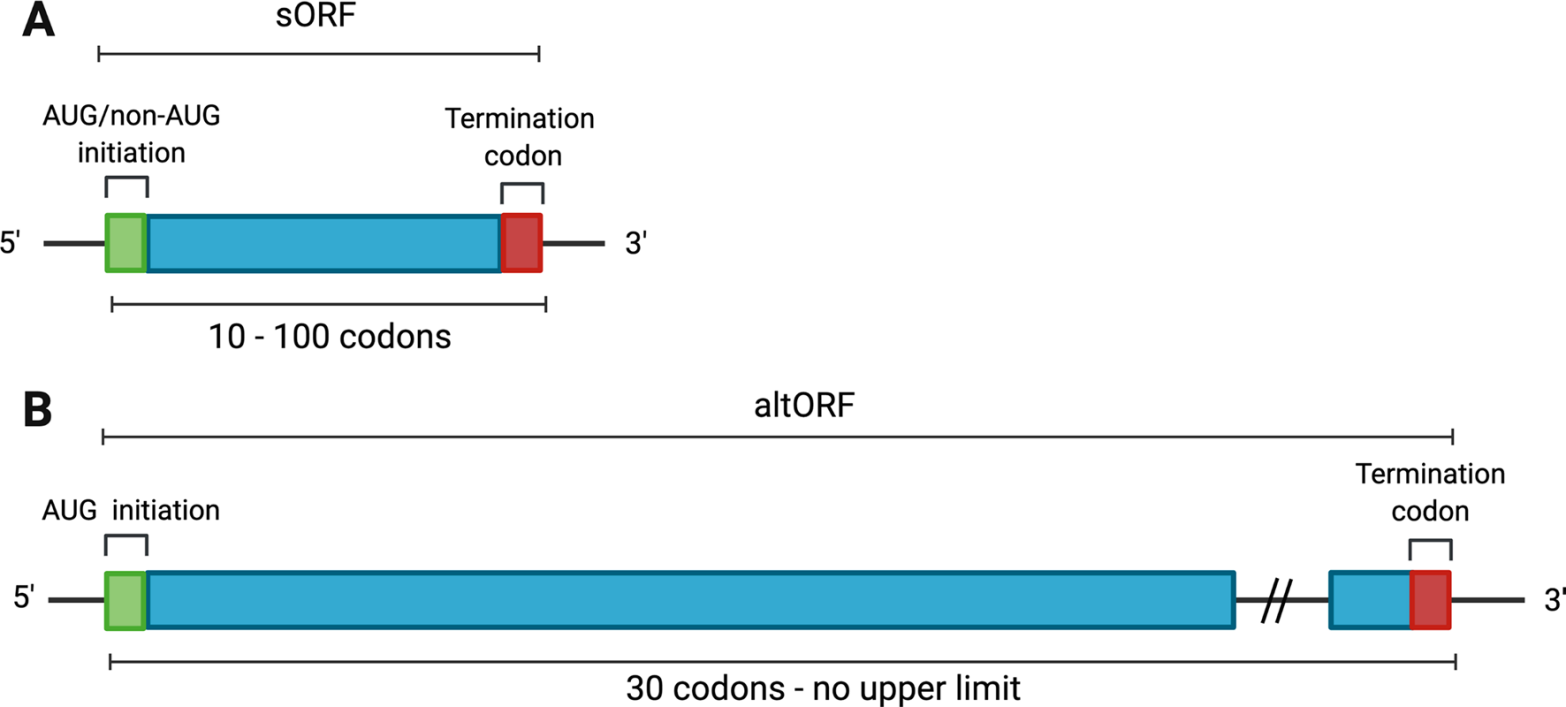
A comparison between sORF and altORF transcripts in terms of length and initiation codons. **A** sORF transcript structure with AUG or non-AUG initiation codons, characterised by its short length of 100 codons after post-transcriptional modifications. **B** altORF transcript structure described with AUG initiation codon, longer than 30 codons and without an upper limit on length, differing from sORFs

Ever since its discovery, sORFs, along with its short lengths, were considered unorthodox, as it could be initiated with AUG as well as non-AUG codons [22–24]. As unconventional ORFs, the definition of sORFs overlaps with another distinctive class of ORFs, known as alternative ORFs (altORFs) (Fig. 1). The altORFs yield transcripts that initiate only with AUG codons and are at least 30 codons, but without an upper length limit [16]. AltORFs were also found to encode proteins, an example being AltMRVI1 from the 3’ UTR of *MRVI1* interacting with BRCA1 in the nucleus [25]. The overlapping definitions of these unconventional ORFs were consequently represented in protein databases, where there is apparent annotation of sORFs under altORFs prior to sole focus of research on sORFs.

## The localities and regulatory functions of sORFs

The 300-base cut-off raises a paradox by contradicting the initial aims of HGP to resolve all ambiguities in the human genome [1]. Post cut-off, there is now a limitation concerning the sORFs and their translated products [4, 20]. There is indefiniteness when it comes to discussing sORFs, and whether they exist putatively or due to random sequence matching to other ORFs. This subsequently leads to obscurity in the methods used for determining microproteins.

**Fig. 2:**
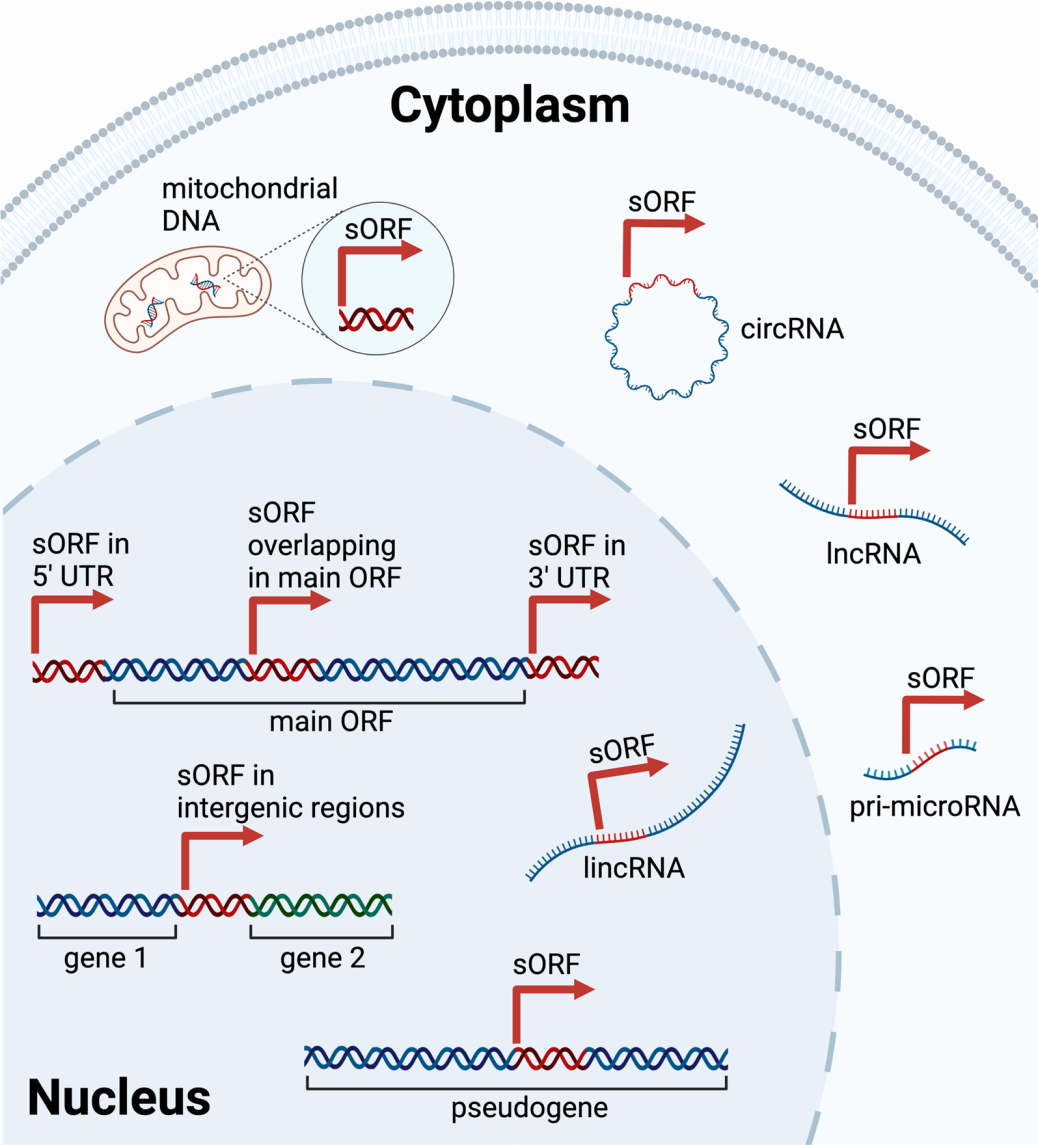
Localities of sORFs in the genome and transcripts. Genomic locations of sORFs include in the 3’ UTR (uORF), 5’ UTR (dORF), overlapping within the main ORF, intergenic regions and pseudogenes. sORF-containing long intergenic non-coding RNA (lincRNA) are also localised in the nucleus. In the mitochondria, sORFs are found in the mitochondrial DNA (mtDNA). In the cytoplasm, sORFs are scattered across different RNA transcripts i.e., circular RNA (circRNA), long non-coding RNA (lncRNA), and pri-microRNA

In continuance from a bulk of research on protein-coding genes, there was simultaneously an exponential increase in non-protein coding elements discovered [2]. By scanning these non-coding regions, researchers have found embedded sORFs that scatter in different genomic locations [26]. The different localities of sORFs are shown in Fig. 2. These locations include the upstream (uORF) and downstream open reading frames (dORF) within the 5’ and 3’ untranslated regions (UTRs) of a gene, even overlapping with the main ORF if the sORF is out of frame [27–31]. Examples of small uORFs include two uORFs in *MDM2*, where the translation products repress the main ORF encoding MDM2 [28]. Other essential genes involved in the developmental process such as *POU5F3* (*Oct4*), *Smad7* and *Nanog* also encode multiple small uORFs, as discovered in zebrafish and human [28, 30]. Whereas, the expression of dORFs was found to enhance the translation of canonical ORFs, where thousands of dORFs have been found translated in human cells and zebrafish embryos [31]. Furthermore, an example of an overlapping sORF is uORF2 in the *ATF4* gene, whereby it represses the transcription of ATF4 under normal conditions [27, 29].This suggests the regulatory role of sORF in post-transcriptional control and translational efficiency [20, 32, 33].

In addition, sORFs are found among pseudogenes and intergenic regions too, with the latter being more difficult to identify due to the high false-negative rates of existing gene finding algorithms [32, 34]. As examples, Kalyana-Sundaram et al*.* (2012) reported the expression of cancer-specific pseudogenes, such as *KLKP1* which encodes a 54-amino acid microprotein in LNCaP, a prostate cancer cell line [35]. Meanwhile, Hanada et al*.* (2007) discovered that 4282 sORFs are located in the intergenic regions, out of 7159 sORFs in *Arabidopsis thaliana* [36]. Apart from genomic DNA, sORFs are encoded by mitochondrial DNA (mtDNA), where the microprotein products have been shown to play roles in muscle and fat metabolism [37]. Besides, sORFs are found embedded in various RNA transcripts previously believed to be non-coding, such as pri-microRNA, circRNA, lincRNA and lncRNA [8, 20, 26, 38–49].

The emergence of new transcripts from genomic regions previously considered non-coding has presented sORFs as a new source of protein-coding genes, consistent with the depiction of an evolutionary mechanism for generating novel polypeptides. With evolutionary conservation, the continuous expression of certain sORFs may provide selective advantages, hence withstanding time and becoming de novo protein-coding genes [8].

## Strategies to detect sORFs with bioinformatics prediction

The need to re-evaluate the coding potentials of sORFs was raised as these minute ORFs were found to be capable of being transcribed and possibly translated. Several methods based on computational analysis, next-generation sequencing (NGS) and mass spectrometry (MS) have been employed to predict the coding potentials, sequences, and identify these sORFs or their products. These measures confirm that such sORFs contain actual coding sequences (CDSs) and produce functional polypeptides; as some bona fide lncRNAs were found to not encode for microproteins, such as XIST, HOTAIR and NEAT1 [50]. To begin with, one must understand the difficulties in detecting microproteins, whereby their smaller sizes (< 100 amino acids; < 20 kDa) necessitates the adaptation of existing laboratory techniques.

Bioinformatic predictions of sORFs proved valuable since it does not cost nearly as much as experimental validations. To differentiate expressed elements based on functionality, several aspects are considered i.e., (i) the conservation of a particular sequence through evolution, and (ii) sequence similarity. Sequence conservation weighs in evolutionary selection, indicating that the sequence remains functionally useful throughout phylogenetic trees [3, 51]. Whereas sequence similarity denotes similar protein motifs or domains aligned over previously identified protein sequences so as to derive coding potentials and potential protein functionalities [4, 17].

Initially, the detection of sORFs was limited by the conservativeness of gene finder algorithms, as only few bioinformatic tools were specifically designed specifically for sORFs. One pioneering sORF prediction tool is sORF finder, which applies coding index (CI) based on nucleotide composition bias in predicting CDSs [52]. This tool successfully enabled the identification of 2376 putative sORFs in the intergenic regions of *Arabidopsis thaliana* [36]*.* From this set of sORFs, Hanada et al*.* (2013) conducted a follow-up study and reported the overexpression of 473 sORFs that were associated with plant morphogenesis [53]. Since then, the training datasets used for such tools have become much larger, realizing higher prediction qualities. Experimental validations of sORFs are now used for homology searches, for ab initio training and for machine learning purposes in the development of better sORF prediction tools [4, 21]. Table 1 provides a list of sORF prediction tools that are applicable to multiple species, and their web addresses. It should be noted that since microproteins are distinct from normal-sized proteins in terms of biophysical properties, some of these tools may not be optimised for the detection of sORFs.

**Table 1:** sORF prediction tools

Prediction tool	References	Website	Description
Coding Non-Coding Identifying Tool (CNIT)	[126]	http://cnit.noncode.org/CNIT/	Distinguishes between coding and non-coding regions based on intrinsic sequence compositions
Coding Region Identification Tool Invoking Comparative Analysis (CRITICA)	[127]	http://rdpwww.life.uiuc.edu/	Analyses nucleotide sequence composition and conservation at the amino acid level
Coding Potential Calculator (CPC)/CPC2	[128, 129]	http://cpc.cbi.pku.edu.cn http://cpc2.gao-lab.org/	Assess protein-coding potential based on important features (ORF size, coverage, integrity); CPC2 improves run speed and accuracy
Coding Potential Predictor (CPPred)	[130]	http://www.rnabinding.com/CPPred/	Predicts the coding potential of RNA transcript
CPPred-sORF	[131]	http://www.rnabinding.com/CPPred-sORF/	Addition of 2 new features from CCPred i.e., GCcount, mRNN-11codons and CUG, GUG start codons
MicroPeptide Tool (MiPepid)	[21]	https://github.com/MindAI/MiPepid	Identifies coding sORFs based on existing microproteins subpopulation set
sORF Finder	[52]	http://evolver.psc.riken.jp/	Identifies sORF with high coding potential based on nucleotide composition bias and potential functional constraint at the amino acid level
smORFunction	[132]	https://www.cuilab.cn/smorfunction/home	Provides function prediction of sORFs/microproteins
miPFinder	[133]	https://github.com/DaStraub/miPFinder	Identifies and evaluates microproteins functionality using information on size, domain, protein interactions and evolutionary origin
PhastCons	[134]	http://compgen.cshl.edu/phast/	Based on conservation scoring and identification of conserved elements
PhyloCSF	[135]	http://compbio.mit.edu/PhyloCSF	Determines a conserved protein-coding region based on formal statistical comparison of phylogenetic codon models
uPEPperoni	[136]	http://upep-scmb.biosci.uq.edu.au/	Specifically for 5’UTR sORFs, based on conservation
AnABLAST	[34]	http://www.bioinfocabd.upo.es/ab/	Identifies putative protein-coding regions in DNA regardless of ORF length and reading frame shifts
Small Peptide Alignment Discovery Application (SPADA)	[137]	https://github.com/orionzhou/SPADA	Homology-based gene prediction programme
Deep Neural Network for coding potential prediction (DeepCPP)	[138]	https://github.com/yuuuuzhang/DeepCPP	Effective on RNA coding potential prediction, spefically sORF mRNA prediction

This table shows prediction tools that can be used for putative sORF detection based on sequence homology and similarity in all genomes. CNIT and CPPred utilises a positive set of normal-sized proteins and may not be optimised for sORF and microprotein detection. CPPre-sORF is an improved version of CPPred for sORF detection. MiPepid, sORF Finder, miPFinder and smORFunction are designed especially for sORF detection, identification, and function prediction. PhastCons, PhyloCSF, SPADA and uPEPperoni utilise conservation analyses for prediction, with the latter designed spefically for sORFs in the upstream region. DeepCPP is based on a deep learning method to evaluate RNA coding potential and demonstrated high performance in sORF data

## Ribosome profiling (RIBO-Seq)

Although the coding potentials of sORFs can be predicted via computational tools, this does not provide sufficient evidence that these sORFs were transcribed and translated. Particularly, many recently discovered putative sORFs were not considered de novo protein-coding genes due to their low levels of evolutionary conservation (e.g., PhyloCSF signals) since these newly found ORFs are evolutionarily very young [54]. The lack of evolutionary constraint renders these sORFs impossible to identify without supporting experimental techniques such as ribosome profiling (RIBO-seq) [54, 55].

**Fig. 3:**
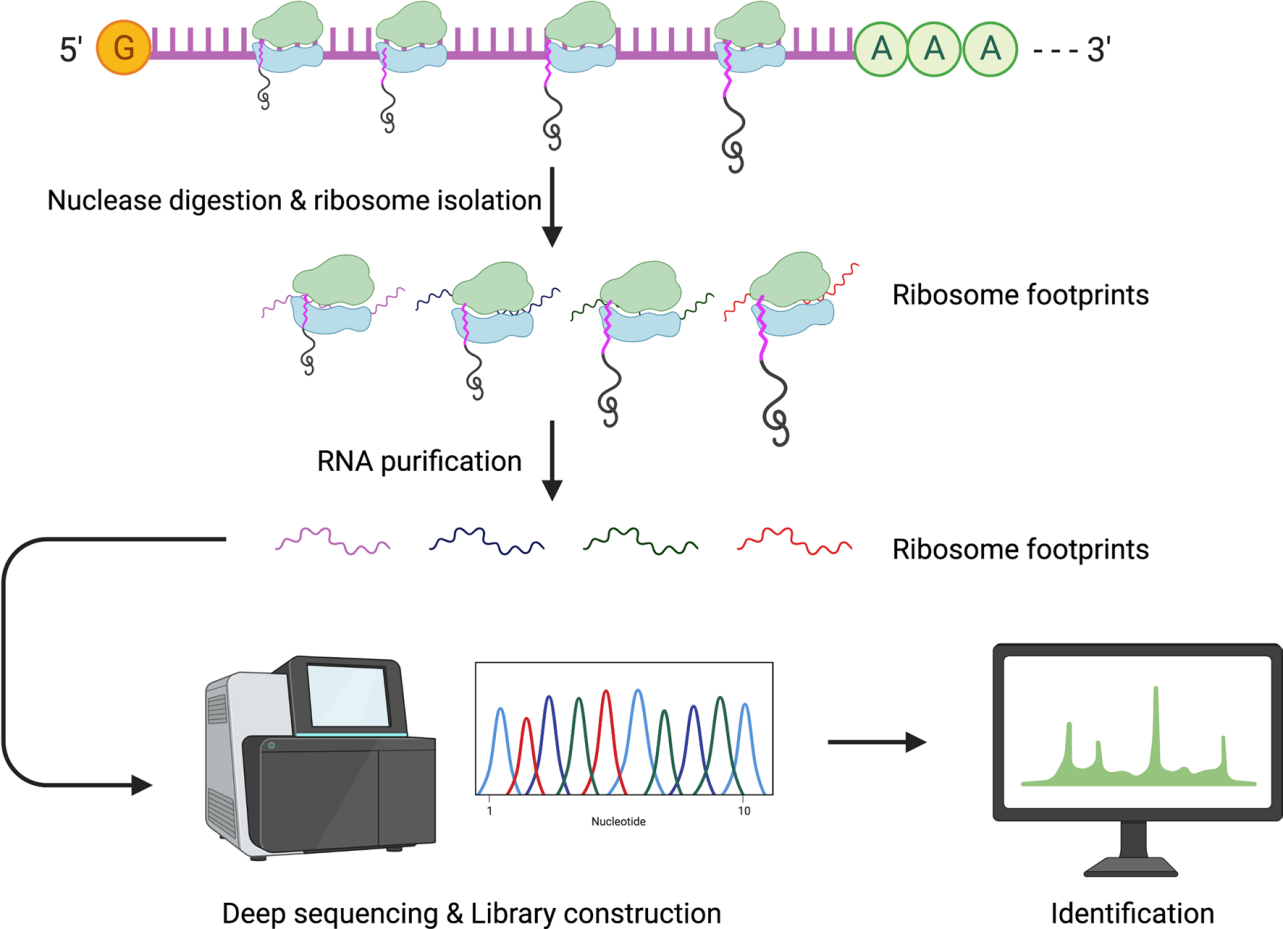
Ribosome profiling process where ribosome footprints are obtained for deep sequencing. Isolation of ribosome-bound mRNAs is conducted through treatment of non-specific nucleases such as RNase I or micrococcal nuclease). Ribosome footprints (showing positioning between start and stop codon of gene) are then used for library generation and deep sequencing. Identification of novel small peptides made possible by isolation of actively translated regions of the transcript, which is directly mapped back to genomic coding regions

Translatomics, the measurement of cellular translational activity, provides not only a snapshot of the translation process, but also on how translation regulates proteome composition. Translatomics experiments were enabled by ribosome profiling or RIBO-Seq, an NGS-based tool developed by Ingolia et al*.* (2009) for measuring ribosome-protected mRNA fragments (Fig. 3) [56]. RIBO-Seq has been applied to identify novel sORFs to explore the protein-coding potentials of RNAs in various models, including mouse embryonic stem cells, budding yeast and *Drosophila* [56–58].

On average, a ribosome can bind and protect 31 nucleotides of mRNA during translation, forming a ribosome footprint. Isolating and identifying ribosome footprints help unveil the translation of polypeptides by systematically recording the exact positions at which translation is halted, after the addition of a chosen protein synthesis inhibitor. Through ribosome footprinting, novel and translatable sORFs can be detected and annotated to their respective genomic coding regions [59, 60]. Treatment with translation inhibitors such as harringtonine or lactimidomycin prior to deep sequencing stalls actively translating ribosomes at the sites of translation initiation [20, 59]. With RIBO-Seq, not only can the translation start sites be identified, but also affinity-based isolation of translating ribosomes is possible, to avoid co-purification of other ribonucleoproteins with the ribosomes [61–63]. Moreover, conventional RIBO-Seq can be modified into Poly-RIBO-Seq (isolation of polysomes), where multiple clusters of ribosomes on a transcript are isolated, providing a more concrete proof of active translation and thereby reducing the number of false positives. Aspden et al*.* (2014) utilised Poly-RIBO-Seq and reported identification of two types of sORF in *Drosophila* in a genome-wide assessment of sORF translation, i.e. (i) longer sORFs (~ 80 amino acids) with resemblance to canonical proteins and (ii) less conserved shorter sORFs (~ 20 amino acids) without functional characterisation from existing bioinformatic pipelines [44].

The most widely used information from ribosome footprints is the 3-nucleotide periodicity, where the codon-wise ribosome movement enables detection of frameshift events and overlapping ORF translations [64, 65]. Besides, the rate of synthesis for a particular peptide could be deduced based on the density of protected fragments obtained. The empirical measurement of protein identity is possible by determining the position of the said footprints [60]. A key advantage of applying RIBO-Seq in the identification of microproteins is the ability to identify sORFs with both AUG and non-AUG initiation codons [22]. Several studies have discovered and confirmed that half of translated sORFs are initiated with a non-AUG codon [23, 24].

Nevertheless, RIBO-Seq must be seen in the light of some limitations. These include experimentally induced distortions due to the need for rapid inhibition of ribosomes to reflect a particular physiological state, thus leading to possible inaccuracies in data collection [59]. Inferring protein synthesis rates from RIBO-Seq builds on the assumption that all ribosomes complete the translation process. However, it would be inaccurate to assume so, since regulated translation pausing and abortion can occur in different physiological conditions, such as starvation [66]. Besides, the transcript capture process by ribosomes can be non-specific and transient whereby no functional polypeptides are produced [67, 68]. The ability of RIBO-Seq to identify protein-coding capacity is limited, as a seminal paper from Guttman et al. raised the idea that ribosome occupancy alone is not a reliable indicator of classifying a transcript as coding or non-coding. Possible explanations include protection of RNA molecule by non-ribosomal RNA–protein complexes, or some of the observed fragments were not from 80S ribosomal footprints [69]. On top of that, some microproteins derived from overlapping ORFs or alternatively spliced transcripts may also avoid detection by RIBO-Seq [70]. Consequently, RIBO-Seq, as useful as it is in providing information on the translation of sORF-containing transcripts, requires another complementary method for further confirming the completed products of sORF translation.

## Mass spectrometry-based approaches

**Fig. 4:**
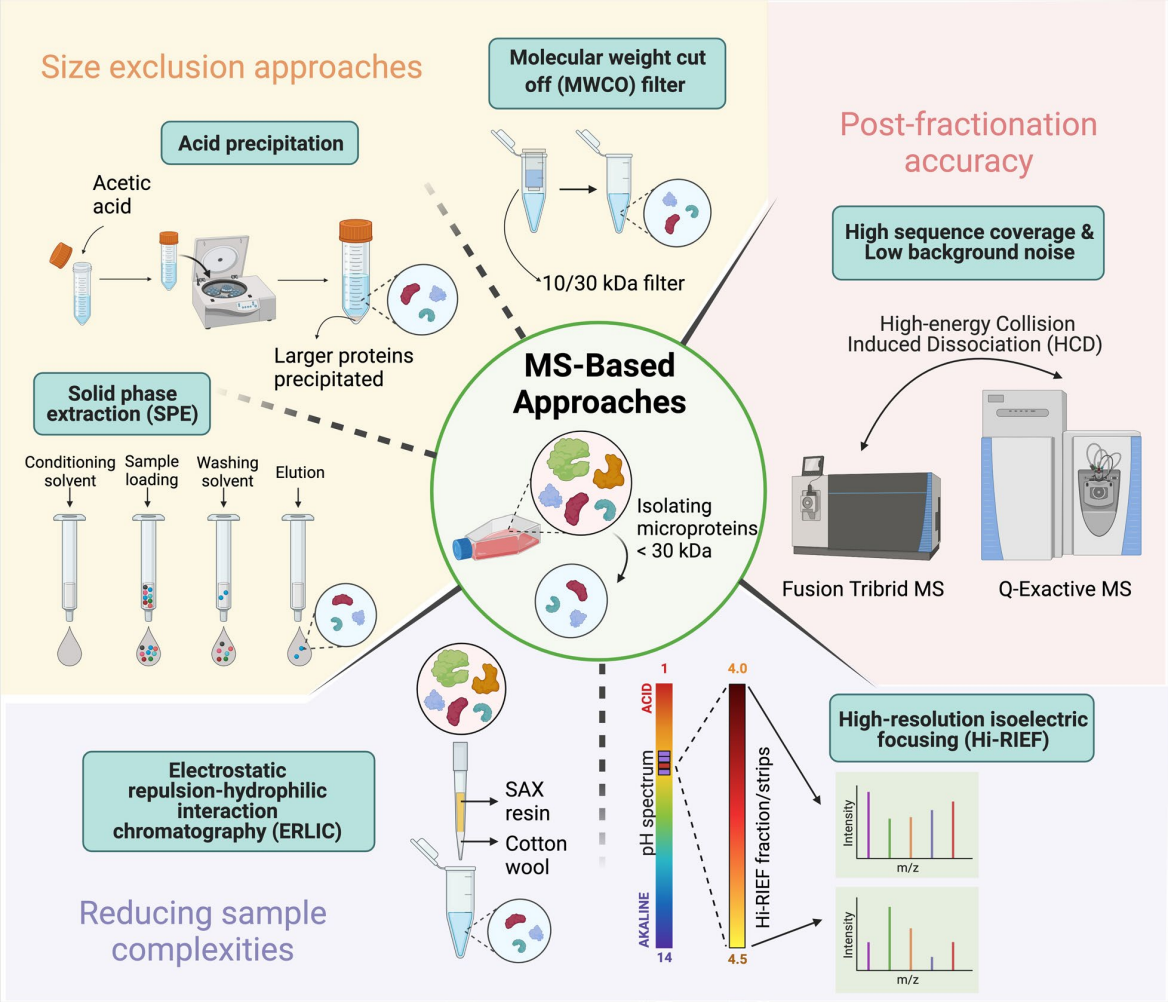
Mass-spectrometry based approaches to isolate microproteins. Sample preparation prior to LC–MS/MS analysis to isolate microprotein species < 30 kDa in size includes size exclusion approaches. Molecular weight cut off filters (MWCOs) can sieve for microproteins depending on the type of filter used i.e., 10 kDa or 30 kDa. Acid precipitation is a common enrichment step for to precipitate larger proteins. Solid phase extraction (SPE) enrichment occurs via reverse-phase C8 cartridges and elutes microproteins of interest. Further methods in reducing sample complexities include electrostatic repulsion-hydrophilic interaction chromatography (ERLIC) and high-resolution isoelectric focusing (Hi-RIEF). ERLIC separates based on charged analytes and utilises SAX resin for strong anion exchange, whereas Hi-RIEF seperates peptides based on their isoelectric points (pI) on a pH gradient gel. Post-fractionation accuracy is dependent on high sequence coverage and low background noise in mass spectra. This can be achieved with using High-energy Collision Induced Dissociation (HCD) on Fusion Tribrid MS or Q-Exactive MS

RIBO-Seq alone does not provide sufficient evidence for the expression of sORF at the protein level although it does demonstrate the translatability of a selected sORF [32, 44, 71–74]. Whereas MS-based proteomics remains indispensable in proteomics for sequencing and quantifying proteins and peptides. Thus, MS strategy can be incorporated in sORF research because such procedure directly analyses the pool of microproteins. Notwithstanding, MS-based profiling of the microproteome requires prior optimisation and modification mainly due to the low abundance and small sizes of microproteins. Notably, a key procedure is to reduce sample complexity using pre-fractionation/enrichment approaches, as shown in Fig. 4.

Size exclusion approaches have been widely employed in peptidomics studies to filter for low-molecular-weight peptides from total lysate [75, 76]. For instance, molecular weight cut off (MWCO) filters are commonly used to retain high molecular weight proteins on the filter, resulting in the enrichment of microproteins in the filtrate. However, Ma et al*.* (2016) had reported that alternative enrichment procedures such as acid precipitation and C8 reverse phase solid phase extraction (SPE) cartridges could result in higher enrichment of microproteins, and therefore a combination of both enrichment procedures was recommended to maximize the recovery of microproteins [75]. Another pre-fractionation measure is electrostatic repulsion-hydrophilic interaction chromatography (ERLIC), that allows charge-driven, orthogonal fractionation of peptides prior to LC–MS/MS [70]. In addition to ERLIC, pre-fractionation with high resolution isoelectric focusing (Hi-RIEF) has also been shown to improve the detectability of microproteins by MS in a highly reproducible manner [77].

To assign peptide sequences with high confidence, high quality MS/MS spectra are crucial, with two main aspects to consider i.e., high sequence coverage and low background noise. To obtain high sequence coverage, Ma et al*.* (2016) compared MS/MS of microprotein-derived peptides between Collision Induced Dissociation (CID) and High-energy Collisional Dissociation (HCD) on Fusion Tribrid MS and Q-Exactive MS [75]. They found that the latter yielded improvements in peptide sequence coverage and lower background noise [75]. Apart from the direct identification and quantification of translated microproteins, MS-based approaches help decipher post-translational modifications (PTMs), such as phosphorylation of microproteins to infer insights in biological functions and signalling pathways [78–80].

Nonetheless, there are several challenges associated with MS-based microproteomics. First, the small size of microproteins render them under-detected by MS due to the low number of tryptic peptides generated [70]. Besides, smaller peptides tend to contain fewer arginine and lysine residues, resulting in non-cleavage or reduced tryptic cleavages. A solution is to replace or to combine trypsin with other proteolytic enzymes, such as Glu-C, Lys-C, Lys-N, Asp-N, Arg-C and chymotrypsin [81–83]. Not to mention, sequential digestion incorporating proteases with complementary cleavage specificities is beneficial for enhancing microprotein identification [84, 85]. Still, microproteins usually lack stable secondary structures, leading to rapid degradation during extraction [86].

Data-dependent acquisition (DDA), the most widely-adopted MS acquisition methods in microproteomics, is prone to under-sampling as MS is limited by sample complexity and sequencing speed [51, 87, 88]. Furthermore, DDA-based detection of microproteins is “amplification-free” and thus limited by the sensitivity and dynamic range of the MS [89]. This leads to a long-standing preferential detection of higher abundance proteins and proteins containing peptides with higher ionisation efficiencies. Conventional bottom-up/shotgun proteomics using DDA is biased towards the detection of proteins with > 10 kDa, which represents more than 90% of the annotated proteome [89]. In addition to this, conventional MS studies using DDA report statistically significant under-representation of experimentally identified small proteins without the inclusion of enrichment procedures for microproteins [89]. The challenge with bottom-up proteomics for microprotein detection lies in several aspects, i.e., (i) during experimental sample preparation, where the lack of necessary cleavage sites in microproteins restricts its digestion; (ii) the need for alternative proteases, due to lack of generating MS-detectable peptides; (iii) under-representation of sORFs in conventional databases, where most gene algorithms apply a length restriction threshold to avoid annotating spurious sORFs; and (iv) in orthodox peptide spectrum matching (PSM) requiring detected microproteins to be matched against two unique peptides for higher confidence in protein identification, whereby microproteins are often only identified by only a single peptide owing to their small sizes [6, 90–93]. This results in an elevated false discovery rate (FDR) in protein identification [92, 93]. Hence, conventional MS should be modified to allow for a more reliable and efficient discovery of small proteins.

In lieu of DDA-based MS detection, targeted proteomics is a promising candidate for a higher confidence identification of microproteins. Selected reaction monitoring (SRM) and data-independent acquisition (DIA) can be used to monitor changes in microprotein expression across different biological samples [87]. In particular, DIA is able to validate, quantify and provide a more complete picture of the microprotein expression as compared to DDA [94, 95]. However, these approaches require specific a priori knowledge of known targets and thus are not suitable for microprotein discovery.

The sequence of a microprotein may closely resemble or overlap with that of motifs derived from larger proteins. In the context of MS-based microprotein discovery, this overlap in the form of shared peptides presents an impediment in protein inference when differentiating between a microprotein and other homologous sequences. In another aspect, microproteins tend to score low in conservation due to their short lengths, which in turn, leads to a high rate of false positives in computational methods [17, 19]. This misalignment of information may also be present in reference protein databases. Consequently, these limitations in MS methods call for an integrated strategy i.e., the proteogenomics approach.

## Proteogenomics approach

Proteogenomics is a comprehensive approach where MS data is coupled to genomic, transcriptomic or translatomic data from the same source, providing an alternative for further validation of low-abundant microproteins [96, 97]. Recent developments in both proteomic and deep sequencing have rapidly established proteogenomics as a reliable technique for studying unexplored or partially sequenced genomes [98–100]. As microproteins fulfil both the criteria of being low-abundant and a relatively new and uncharacterised class of proteins, applying proteogenomics techniques is a reasonable and coherent measure.

This approach involves generating a customised protein sequence database from genomic and transcriptomic sequences to be matched against MS spectra. By compiling predicted, novel peptide sequences and their variants, proteogenomics also refines protein sequence databases. Several proteogenomic studies had mapped MS spectra to RNA-seq data for detecting unannotated sORFs [76, 101, 102]. However, this strategy often suffers from reduced sensitivity and reliability because of the inflated search space. This is because a bloated protein database that is in silico translated from RNA-Seq data also comes with increased false positive peptide-spectral matches [70]. Proposed measures to remedy this problem includes incorporating (i) protein fractionation, (ii) in silico filters and (iii) RIBO-Seq data instead of RNA-Seq data, so that higher sORF-specificity and selectivity can be achieved [103, 104].

## Microprotein databases

Until now, the number of sORFs and microproteins which they encode have been accumulating. In Table 2, we compiled several publicly available repositories specialized for sORFs. Incorporating a computational pipeline to corroborate with experimental data obtained would boost the credibility when characterising annotated and unannotated small peptides. A substantial number of studies have integrated this combinatorial approach in identify microproteins, proving its advantage when addressing the technical issues on validating coding sORFs [51, 71, 73, 102, 105, 106].

**Table 2:** Online repositories tailored for sORF identification

Database	References	Website	Type	Description
sORFs.org	[14]	http://www.sorfs.org	sORF repository	Obtains experimental data from RIBO-seq with conservation analyses and rescanning MS data from PRIDE for updated small peptide validation
SmProt	[109]	http://bioinfo.ibp.ac.cn/SmProt/	sORF repository	Database on small proteins specifically from lncRNA, obtains data from RIBO-seq, literature mining and MS data, integrates conservation analyses
OpenProt	[111, 112]	https://www.openprot.org/	altORF resource	Contains information on protein isoforms and altORFs with experimental evidence, intergrates RIBO-seq, MS, conservation analyses and functional domains
ARA-PEPs	[108]	http://www.biw.kuleuven.be/CSB/ARA-PEPs	sORF repository	Repository of putative sORF-encoded peptides specifically in *Arabidopsis thaliana*, data obtained from in-house Tiling arrays and RNA-seq data
PsORF	[107]	http://psorf.whu.edu.cn/	sORF repository	Database of sORF across different plant species, incorporating genomic, transcriptomic, RIBO-Seq and MS data
MetamORF	[110]	http://metamorf.hb.univ-amu.fr/	sORF repository	A repository of unique sORFs in *H. sapiens* and *M. musculus* genomes by experimental and computational methods
nORFs.org	[113]	https://norfs.org/	novel ORF (nORF) repository	Provides aggregated information from databases such as sORFs.org, OpenProt and OpenCB

This table shows the databases available publicly for sORF identification. sORFs.org and OpenProt evaluate protein sequence identity based on BLASTp score, whereas SmProt provides a BLAST alignment search for manual evaluation of protein sequence identity. OpenProt annotates sORFs but under the label of altORFs that are longer than 30 codons and originating from ncRNAs, pseudogenes or has multiple ORFs per transcript, hence the limits set during search identification should be noted. ARA-PEPs were developed specifically from *A. thaliana* sORF experimental data, and PsORF aimed to store a more complete record of plant sORF. A large bulk of both MetamORF and nORFs.org data was obtained from sORFs.org and OpenProt. nORFs.org provides additional protein sequence viewer, OpenCB variants and customises annotation metrics functions

A few of the databases mentioned in Table 2 are specifically tailored for storing sORF information, such as sORFs.org, SmProt, ARA-PEPs, PsORF and MetamORF. However, between these databases, PsORF and ARA-PEPs are resources specially for plants, with the latter storing sORF data exclusively for *Arabidopsis thaliana* [107, 108]. For databases with higher coverage over sORFs datasets in multiple organisms, sORFs.org and SmProt are more suitable since they store sORF data over a range of organisms, such as human, mouse, rat, zebrafish, nematode and fruit fly [14, 109]. In addition to that, SmProt also saves sORF data from bacteria and yeast [109]. For detailed query, sORFs.org incorporates a Biomart function, where one can tailor the search for microproteins by customising their queries according to species, chromosome number, start codons and sORFs attributes [14]. On the other hand, MetamORF is more limited in the sense that it only contains sORF data from *Homo sapiens* and *Mus musculus*, but its data has been experimentally and computationally verified, where one can browse the database according to gene locus, ORF, and transcript [110]. For OpenProt, since it is a large database, sORFs are stored under the heading of altORFs, where there is annotation of sORFs within ncRNA and pseudogene transcripts, hence some data downloaded from OpenProt in FASTA files may contain sequences longer than 100 codons [111, 112]. Finally, nORFs.org compiled data from sORFs.org and OpenProt, whilst also incorporating genomic information from OpenCB. This allows for nORFs.org to complement information from both databases and incorporate OpenCB variants in its database [113]. Choosing which database to use when annotating experimental microproteins is highly dependent on the sample used, as well as the variety of information and query method that is provided by each database.

## Validation of microproteins and their biological functions

Whilst the aforementioned methods are efficient at detecting microproteins, a validation step is required to elucidate the exact biological functions of these identified microproteins. However, since it is unnecessarily complicated to validate all possible microproteins, a rational step is to narrow down these sORF candidates into a simplified list [96]. One strategy is to analyse differential expression data of the transcripts or proteins. As an example, Cao et al*.* (2020) investigated 16 potential microproteins from leukaemia cell lines K562 and MOLT4; and found that 4 out of 16 of these microproteins were differentially expressed, demonstrating their potential roles in leukaemia [24]. Another approach is to focus on specific genomic regions which are likely to possess high coding potentials, and thus capable of coding for microproteins of interest. As demonstrated by Lee, Kim and Cohen (2016) and H. Lu et al*.* (2019), a study was conducted on a selected mtDNA genomic region which encodes MOTS-c, a microprotein which plays a role in muscle and fat metabolism [37, 114].

To explore the biological roles of microproteins, one must first understand their modes of actions. Microproteins exert their cellular functions either by forming a complex with larger canonical proteins, or by acting autonomously [10, 96]. Slavoff et al*.* (2013) proposed that microproteins need to exist at biologically relevant concentrations to exert their physiological function [23]. Thus, by logic, genome-wide CRISPR/Cas9-based screens would be advantageous in detecting the extent to which these microproteins have an influence on the phenotype [45, 73, 115–118]. For direct observation of the targeted microprotein, loss-of-function (e.g., knockdown, knockout) or gain-of-function (e.g., overexpression or activation) assays can be performed and the function of the microprotein can be deduced based on the resulting phenotype. The scale of how altered the phenotype is from the wildtype can infer insights on how influential the microprotein is in that process, and at which concentrations they produce significant effects.

To elaborate, Stein et al*.* (2018) validated the function of a 56-amino-acid microprotein mitoregulin (Mtln) in supporting mitochondrial super-complexes and respiratory efficiency [116]. They reported disturbances in mitochondrial respiratory super-complex formation, reduced fatty acid oxidation, TCA cycle enzymes and calcium ion retention capacity in Mtln-knockout mice models, whereas Mtln overexpression in HeLa cells led to increased mitochondrial respiratory and calcium ion buffering capacities, whilst decreasing generation of reactive oxygen species [116]. Another study came from in-vivo work by Matsumoto, Clohessy and Pandolfi (2017) [115]. They uncovered a novel microprotein SPAR that played a role in regulation of mTORC1 recruitment that resulted in reduced muscle regeneration, through Spar-deficient mice models [115]. In addition, Lu et al*.* (2019) reported functional characterisation of lncRNA-encoded microprotein UBAP1-AST6 in A549 lung cancer cell line by overexpression and knockout models, where the prior significantly promoted cell proliferation and clonogenic property [102]. Nonetheless, since microproteins may reside in lncRNA instead of mRNA, this presents an obstacle when applying CRISPR/Cas9 editing, as CRISPR targeting disrupts the expression of both lncRNA and mRNA, impeding clear interpretation of genome editing data. One solution would be to selectively block the expression of microprotein by mutating or knocking in the start codon, while allowing lncRNA expression at the same time [86, 118].

On the contrary, another application for CRISPR/Cas9 is to knock-in an epitope sequence into the native sORF sequence so that the expression and localization of said sORF sequence can be monitored by corresponding antibodies that bind to the epitope tags. [96]. Choices of epitopes include FLAG, APEX or fluorescent proteins and split-fluorescent proteins, among many others, depending on which characteristic of the microprotein that is being scrutinised [24, 45, 119–121]. Apart from assessing the expression levels of tagged proteins, antibody capture of epitope tags can be applied to harvest *bona-fide* prey proteins that interact in a specific manner with the epitope-tagged baits. This form of protein–protein interaction (PPI) assay is named co-immunoprecipitation (coIP) or affinity purification (AP) [120]. CoIP can be applied to functionally validate microproteins, based on the understanding that like most proteins, microproteins exert their functions via the formation of microprotein-protein assemblies. Thus, by co-purifying and identifying bona-fide protein binding partners, the functions of these microproteins can subsequently be elucidated based on the functions or the pathways of the co-purified partners, akin to guilt-by-association. The working principles and the different MS-based strategies for elucidating PPIs have recently been reviewed in detail by Low et al. so it will not be discussed further here [122]. In the context of microproteins, Rodrigues et al*.* (2018) performed coIP with FLAG-GFP transgenic plants and confirmed microprotein miP1a is a part of a DNA binding complex in *FT* promoter, regulating the floral transition in *Arabidopsis* [123]. In another study, the interaction between HA-tagged micropeptide myoregulin (MLN) and membrane pumps SERCA1, SERCA2a and SERCA2b on sarcoplasmic reticulum (SR) was visualised through coIP of its stable complex, thus proving MLN’s role in regulating muscle performance by impeding uptake of calcium ions into the SR [45]. Furthermore, the cytoprotective property of mitochondrial-derived peptide humanin (HN) was demonstrated through coIP of HN-GFP with pro-apoptotic protein BimEL, leading to the conclusion that HN has the capacity to inhibit BimEL-induced activation of Bak and thus inhibiting apoptosis [124]. These studies show the usefulness of coIP in validating microprotein functions, based on the logic of guilt-by-association.

## Conclusions

In the last two decades, sORFs and microproteins represent an expanding landscape, with multiple technological innovations advancing the speed of their discovery and annotations. Exploration of this part of the genome that was disregarded have yielded results in terms of sORF transcription and microprotein functionality. The current measures in identification and validation were successful in unveiling the microproteome, yet progress must be made to address the caveats of these techniques. Functional characterisation of existing microproteins remains to be improved, and the investigation for small peptides in new sample types. There has been some exploration into the potential of microproteins as therapeutic targets. For instance, Ontak (denileukin) was developed as toxins, targeted for tumour-specific microproteins in the treatment for T cell lymphoma [125]. However, through exhaustive literature mining, it appears that this field is not yet extensively studied, thus providing a lead on where future research could perhaps focus on. Previously neglected in the genome, further progress in this field would shed light on sORF and microproteins, with the opportunity to raise new knowledge on a hidden sub-proteome and its role in biological function.

## Data Availability

Not applicable.

## Abbreviations

AltORF: Alternate open reading frame
AP: Affinity purification
CDS: Coding sequences
CI: Coding Index
CID: Collision induced dissociation
circRNA: Circular ribonucleic acid
CoIP: Co-immunoprecipitation
CRISPR/Cas 9: Clustered regularly interspaced short palindromic repeats and CRISPR-associated protein 9
DDA: Data-dependent acquisition
DIA: Data-independent acquisition
DNA: Deoxyribonucleic acid
dORF: Downstream open reading frame
ER: Endoplasmic reticulum
ERLIC: Electrostatic repulsion-hydrophilic interaction chromatography
FANTOM: Functional ANnoTation Of the Mammalian Genome Consortium
GFP: Green fluorescent protein
HCD: High-energy collisional dissociation
HGP: Human Genome Project
Hi-RIEF: High Resolution Isoelectric Focusing
LC–MS/MS: Liquid Chromatography with Tandem Mass Spectrometry
lincRNA: Long intergenic non-coding ribonucleic acid
lncRNA: Long non-coding ribonucleic acid
pri-microRNA: Precursor micro-ribonucleic acid
mRNA: Messenger ribonucleic acid
MS: Mass spectrometry
MS/MS: Tandem mass spectrometry
mtDNA: Mitochondrial deoxyribonucleic acid
MWCO: Molecular weight cut off
ncRNA: Non-coding ribonucleic acid
NGS: Next generation sequencing
NHEJ: Non-homologous end joining
ORF: Open reading frame
PhyloCSF: Phylogenetic codon substitution frequencies
PPI: Protein–protein interaction
PTM: Post translational modifications
RIBO-Seq: Ribosome profiling
RNA: Ribonucleic acid
RNA-Seq: Ribonucleic acid sequencing
SEP: Short open reading frame-encoded proteins
smORF: Small open reading frame
sORF: Short open reading frame
SPE: Solid phase extraction
SR: Sarcoplasmic reticulum
TCA: The citric acid cycle
uORF: Upstream open reading frame
UPR: Unfolded protein response
UTR: Untranslated region
